# Clustered Breeding Sites: Shelters for Vector-Borne Diseases

**DOI:** 10.1155/2018/2575017

**Published:** 2018-07-09

**Authors:** J. C. A. Dias, L. H. A. Monteiro

**Affiliations:** ^1^Universidade Presbiteriana Mackenzie, PPGEEC, São Paulo, SP, Brazil; ^2^Universidade de São Paulo, Escola Politécnica, São Paulo, SP, Brazil

## Abstract

Here, the propagation of vector-borne diseases is modeled by using a probabilistic cellular automaton. Numerical simulations considering distinct spatial distributions and time variations of the vector abundance are performed, in order to investigate their impacts on the number of infected individuals of the host population. The main conclusion is as follows: in the clustered distributions, the prevalence is lower, but the eradication is more difficult to be achieved, as compared to homogeneous distributions. This result can be relevant in the implementation of preventive surveillance measures.

## 1. Introduction

Contagious diseases transmitted by insects remain a serious public-health problem in many countries. For instance, in the past few years, the Brazilian people have been infected by Chagas disease [1], chikungunya, dengue, zika [2], leishmaniasis [3], malaria [4], and yellow fever [5].

The vector-borne pathogen transmission began to be mathematically analyzed in 1908 by Ross [6], who formulated the so-called “mosquito theorem.” According to this theorem, malaria would be naturally eradicated from a region if the mosquito abundance was reduced below a critical value in such a region. In this seminal work, however, the spatial dimension of this region was not explicitly taken into account.

Theoretical investigations on the spread of vector-borne diseases based on cellular automaton (CA) have been carried out [7–14]. In CA models, the spatial features of the host and vector populations, such as mobility patterns and heterogeneities, can be naturally taken into consideration in the CA lattice and in the rules of state transition.

Here, in our CA model, each cell of the CA lattice is occupied by one individual of the host population and in each cell there may be an amount of vectors. At each time step *t*, each individual is in one of three states: susceptible (*S*), infected (*I*), or recovered (*R*). The state transitions between the time steps *t* and *t* + 1 of this SIR-type epidemic model are driven by probabilistic rules. The goal is to examine the influence on the infected host group of distinct spatial distributions and time variations of the vector abundance, by running computer simulations.

This paper is organized as follows. In Section 2, the CA model is described. In Section 3, the results obtained from numerical simulations are presented. In Section 4, the relevance of the results for disease-prevention campaigns is discussed.

## 2. The CA Model

In our CA model, the host population lives in a square lattice with *n* × *n* = *n*^2^ = *N* cells. To eliminate edge effects, the top edge of the lattice contacts the bottom edge and the left edge contacts the right edge (thus, a three-dimensional torus is formed from this two-dimensional lattice). Each cell is occupied by one individual and each individual is in contact with its eight surrounding neighbors, which is usually known as Moore neighborhood of unit radius [15]. Note that, due to the boundary conditions chosen for the CA lattice, all individuals have the same number of neighbors. In addition, suppose that the amount of vectors associated with the *j*th cell is *v*_*j*_. Therefore, the total amount of vectors in the CA lattice is *V* = ∑_*j*=1_^*N*^*v*_*j*_.

The time evolution of the proposed SIR model is ruled by the following set of probabilities of state transition (for similar models, see, for instance, [16, 17]). At each time step *t*, the probability of a *S*-individual being infected is given by *P*_*infection*_ = 1 − *e*^−*μη*^, in which *μ* is the sum of *v*_*j*_ considering the eight surrounding neighbors and *η* is number of *I*-neighbors. Note that *P*_*infection*_ = 0 if *μ* = 0 and/or *η* = 0; hence, the transition *S* → *I* between the time steps *t* and *t* + 1 cannot occur if there are not vectors and/or infected individuals in the neighborhood of such a *S*-individual. Note also that *P*_*infection*_ monotonously increases with *μ* and/or *η*. For “high” values of *μη*, then *P*_*infection*_≃1.

An *I*-individual has probability *P*_*cure*_ per time step of becoming cured and permanently protected against the infection; if not cured, then this *I*-individual has probability *P*_*death*−*I*_ per time step of dying (due to the disease). A *R*-individual has probability *P*_*death*−*R*_ per time step of dying (for other causes). Assume that when *I* and *R*-individuals perish, *S*-individuals replace them. Consequently, the total number of individuals *N* remains constant, which is a convenient assumption for modeling host populations in which the deaths are balanced by the births. Notice that the probabilities *P*_*cure*_, *P*_*death*−*I*_, and *P*_*death*−*R*_ correspond to the state transitions *I* → *R*, *I* → *S*, and *R* → *S*, respectively. The states of all individuals are simultaneously updated throughout a simulation [18].

In many models, the vector population is divided into infected and noninfected subgroups [7–9, 11–14, 19, 20]. In our model, this division is not made; only the vector abundance matters. The rationale for this simplifying assumption is that the higher the amount of transmitters, the higher the probability of an *I*-neighbor being bitten and indirectly infecting a *S*-individual.

In short, the parameters of the model are the probabilities *P*_*cure*_, *P*_*death*−*I*_, and *P*_*death*−*R*_, the total number of individuals *N*, the total amount of vectors (for instance, mosquitoes) *V*, the spatial distribution, and the time variation of *v*_*j*_.

The following spatial distributions are considered in the simulations:

- Uniform distribution: in each cell of the CA lattice, *v*_*j*_ = *v* = c*onstant*.
- Random distribution: in each cell of the CA lattice, there is a 50% chance of *v*_*j*_ = 2*v* and a 50% chance of *v*_*j*_ = 0.
- Column distribution: in each cell of the *j*th-column, *v*_*j*_ = 2*v* if *j* is even and *v*_*j*_ = 0 if *j* is odd.
- One-cluster distribution: in each cell of a region *n*/2 × *n*/2 of the CA lattice, then *v*_*j*_ = 4*v*; outside this region, *v*_*j*_ = 0.
- Two-clusters distribution: in each cell of two regions *n*/4 × *n*/2, then *v*_*j*_ = 4*v*; outside these regions, *v*_*j*_ = 0. These two regions are horizontally separated by *n*/4 cells.
- Four-clusters distribution: in each cell of four regions *n*/8 × *n*/2, then *v*_*j*_ = 4*v*; outside, *v*_*j*_ = 0. These clusters are horizontally separated by *n*/8 cells.

Note that, in all distributions, *V* = *Nv*; thus, the total amount of vectors is the same. Therefore, only the impact of the geographical spread of vectors is evaluated.

We also consider the following time dependencies of *v*_*j*_:

- Time-invariant function: the value of *v*_*j*_ in each cell is not altered during the simulation; therefore, *V* is kept fixed.
- Periodic function: *v*_*j*_ oscillates between two numbers with period *T*. In this case, *v*_*j*_ is kept fixed for *T*/2 time steps, then it is reduced to *qv*_*j*_ (with *q* < 1) for the next *T*/2 time steps, then it returns to the original value *v*_*j*_ for the next *T*/2 time steps, and so on. Thus, the total amount of vectors periodically varies between *V* and *qV*. This variation can be a consequence of seasonal oscillations of climatic variables, such as temperature and humidity [21].

Also, there is no migration of vectors on the lattice; that is, it is supposed that the vectors can move only a short distance from their breeding sites [22, 23].

## 3. Simulation Results

**Figure 1:**
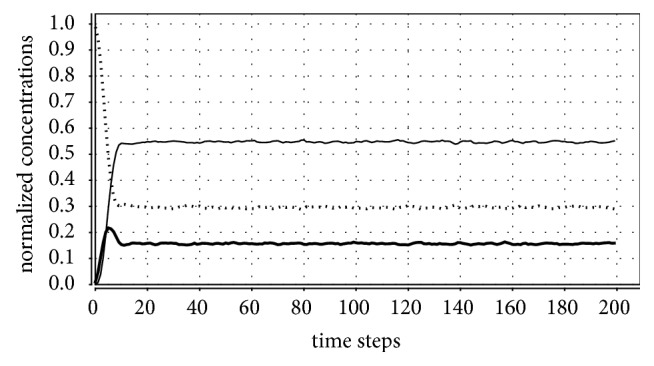
Time evolution of *S*(*t*)/*N* (dotted line), *I*(*t*)/*N* (thick line), and *R*(*t*)/*N* (thin line) from *S*(0)/*N* = 0.99, *I*(0)/*N* = 0.01, and *R*(0)/*N* = 0. Parameter values: *P*_*cure*_ = 70%, *P*_*death*−*I*_ = 40%, *P*_*death*−*R*_ = 20%, and *n* = 200, time-invariant uniform distribution with *v* = 0.1. In this case, the disease endemically persists at a constant level (around 0.1565, as shown in Table 1).

Computer simulations were performed by taking: *P*_*cure*_ = 70%, *P*_*death*−*I*_ = 40%, *P*_*death*−*R*_ = 20%, *n* = 200 (hence, *N* = 40000), and *v* = 0.1, 0.15, and 0.2 (therefore, *V* = 4000, 6000, and 8000, respectively). When time variation in *v*_*j*_ is considered, *V* = 4000, *q* = 9/200, and *T* = 10 or 30. Simulations with other parameter values were performed, but the results were qualitatively the same as reported below. In all simulations, the initial condition (at *t* = 0) is *S*(0)/*N* = 99%, *I*(0)/*N* = 1%, and *R*(0)/*N* = 0%. The asymptotical solution, however, does not depend on this starting point (that is, the attractor is globally asymptotically stable).  Figure 1 illustrates a simulation with 200 time steps in which an endemic solution is reached. In this figure, *S*(*t*)/*N* is represented by a dotted line, *I*(*t*)/*N* by a thick line, and *R*(*t*)/*N* by a thin line.

Table 1 shows the endemic steady-states reached for *v*_*j*_ = constant. For each *V*, the average value of *I*(*t*)/*N* obtained in the last 100 time steps is given. Observe that the percentage of infected individuals grows with *V*, as intuitively expected. For the clustered distributions, this percentage increases with the number of clusters. The values found for uniform, random, and column distributions are similar to each other. The values found for the three clustered distributions are also similar to each other, but they are smaller than those found for the three homogeneous distributions. Obviously, the number of infected individuals can be increased, for instance, by reducing *P*_*cure*_ and/or *P*_*death*−*I*_ [18].

Inspired by the mosquito theorem, another set of simulations was run to determine the critical number of vectors *V*_*critical*_ below which the disease disappears. For values of *V* below the ones shown in Table 2, the infection was naturally eradicated in 10 consecutive simulations. These critical values were numerically found by varying *V* with a step size of 100. Observe that these two tables lead to a surprising conclusion: the prevalence in clustered distributions is lower than in homogeneous distributions; however, clustered distributions impair disease eradication. That is, clustered distributions require a greater effort to eliminate the disease than homogeneous distributions, in which the prevalence is higher! This result should be taken into account in the planning of public-health interventions.

**Figure 2:**
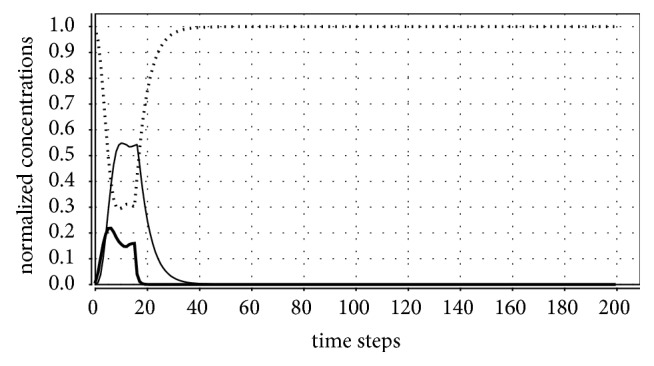
Parameter values: *P*_*cure*_ = 70%, *P*_*death*−*I*_ = 40%, *P*_*death*−*R*_ = 20%, *n* = 200, periodic uniform distribution with *v* = 0.1, *q* = 9/200, and *T* = 30 (thus, *V* oscillates between 4000 and 180 with period 30). In this case, the disease naturally disappears (that is, *I*(*t*) → 0 as the time passes).

**Figure 3:**
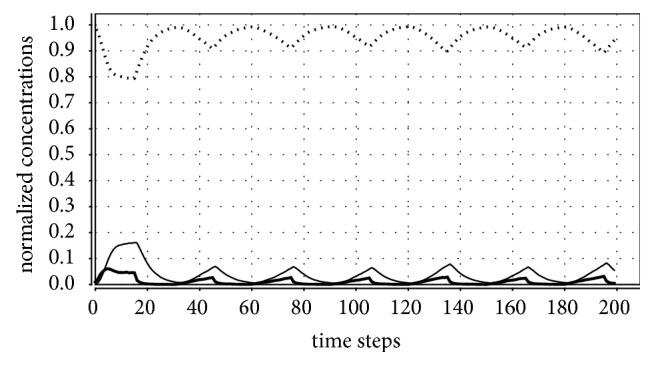
Parameter values: *P*_*cure*_ = 70%, *P*_*death*−*I*_ = 40%, *P*_*death*−*R*_ = 20%, *n* = 200, periodic one-cluster distribution with *v* = 0.1, *q* = 9/200, and *T* = 30. In this case, the disease prevalence tends to a periodic oscillation.

Table 3 presents the number of eradications in 100 simulations for seasonal variation of the amount of vectors. In these simulations, *V* periodically oscillates between 4000 and 180 with period *T* = 10 or 30. Figures 2 and 3 illustrate eradication and persistence of the disease, respectively. These simulations show that the more the vectors are clustered, the lower the number of cases in which the disease disappears. Observe that, for *T* = 10, the disease endemically persists only in the clustered distributions (it is always eradicated in the three homogeneous distributions); for *T* = 30, the disease is always eliminated in the homogeneous distributions and, for the clustered distributions, the number of eradications grows with the number of clusters.

## 4. Conclusions

The influence of spatiotemporal heterogeneities of vectors on the propagation of vector-borne diseases has been experimentally [24, 25] and theoretically [8, 26] analyzed. Here, our simplistic epidemic model reveals that the more the vectors are clustered in a given region, the lower is the prevalence; however, the greater must be the effort to eradicate the disease from such a region.

Reducing the vector population is one of the key methods employed to control the transmission of vector-borne diseases [7, 8, 11–13, 20]. Our simulations suggest that vector-control programmes should take into consideration the spatial distribution of vectors and not only the disease prevalence and/or the vector population size. A decrease in the number of infections can also be achieved by restricting the mobility of the host population in the infected region [9, 11, 14, 19], but this surveillance measure may be difficult to implement in practice.

Big cities are shelters for directly transmitted infections [16, 27]. In agreement with other studies [11, 24], this work proposes that the main focus of vector-control programmes should be to eliminate the large clustered breeding sites of infected big cities, because these sites are shelters for vector-borne diseases.

## Figures and Tables

**Table 1:** Normalized infected population in steady state as a function of *V* for six time-invariant distributions.

distribution	*V* = 4000	*V* = 6000	*V* = 8000
uniform	0.1565 ± 0.0004	0.1667 ± 0.0004	0.1712 ± 0.0003

random	0.1511 ± 0.0005	0.1626 ± 0.0004	0.1679 ± 0.0003

column	0.1480 ± 0.0004	0.1607 ± 0.0004	0.1667 ± 0.0003

1-cluster	0.0455 ± 0.0001	0.0458 ± 0.0001	0.0459 ± 0.0002

2-clusters	0.0462 ± 0.0002	0.0466 ± 0.0001	0.0466 ± 0.0002

4-clusters	0.0476 ± 0.0002	0.0480 ± 0.0002	0.0482 ± 0.0002

**Table 2:** Critical amount of vectors *V*_*critical*_ for six time-invariant distributions.

distribution	*V*_*critical*_
uniform	1200

random	1000

column	1000

1-cluster	300

2-clusters	300

4-clusters	300

**Table 3:** Number of eradications in 100 simulations in function of *T* for six seasonal distributions.

distribution	*T* = 10	*T* = 30
uniform	100	100

random	100	100

column	100	100

1-cluster	0	18

2-clusters	0	41

4-clusters	0	82

## Data Availability

The data used to support the findings of this study are available from the corresponding author upon request.

## References

[1] Freitas E. C., de Fátima Oliveira M., de Barros Vasconcelos A. S. O. (2017). Analysis of the seroprevalence of and factors associated with chagas disease in an endemic area in Northeastern Brazil. *Journal of the Brazilian Society of Tropical Medicine*.

[2] Mota M. T. D. O., Terzian A. C., Silva M. L. C. R., Estofolete C., Nogueira M. L. (2016). Mosquito-transmitted viruses – the great Brazilian challenge. *Brazilian Journal of Microbiology*.

[3] de Carvalho I. P. S. F., Peixoto H. M., Romero G. A. S., de Oliveira M. R. F. (2017). Cost of visceral leishmaniasis care in Brazil. *Tropical Medicine & International Health*.

[4] Recht J., Siqueira A. M., Monteiro W. M., Herrera S. M., Herrera S., Lacerda M. V. G. (2017). Malaria in Brazil, Colombia, Peru and Venezuela: Current challenges in malaria control and elimination. *Malaria Journal*.

[5] Possas C., Martins R. M., de Oliveira R. L., Homma A. (2018). Urgent call for action: Avoiding spread and re-urbanisation of yellow fever in Brazil. *Memórias do Instituto Oswaldo Cruz*.

[6] Ross R. (1908). *Report on the Prevention of Malaria in Mauritius*.

[7] Botari T., Alves S. G., Leonel E. D. (2011). Explaining the high number of infected people by dengue in Rio de Janeiro in 2008 using a susceptible-infective-recovered model. *Physical Review E: Statistical, Nonlinear, and Soft Matter Physics*.

[8] Cissé B., El Yacoubi S., Gourbiére S. (2016). A cellular automaton model for the transmission of Chagas disease in heterogeneous landscape and host community. *Applied Mathematical Modelling: Simulation and Computation for Engineering and Environmental Systems*.

[9] Dommar C. J., Lowe R., Robinson M., Rodó X. (2014). An agent-based model driven by tropical rainfall to understand the spatio-temporal heterogeneity of a chikungunya outbreak. *Acta Tropica*.

[10] Gerardi D. O., Monteiro L. H. A. (2011). System identification and prediction of dengue fever incidence in Rio de Janeiro. *Mathematical Problems in Engineering*.

[11] de Castro Medeiros L. C., Castilho C. A. R., Braga C., de Souza W. V., Regis L., Monteiro A. M. V. (2011). Modeling the dynamic transmission of dengue fever: Investigating disease persistence. *PLOS Neglected Tropical Diseases*.

[12] Pereira F. M., Schimit P. H. T. (2018). Dengue fever spreading based on probabilistic cellular automata with two lattices. *Physica A: Statistical Mechanics and its Applications*.

[13] Santos L. B., Costa M. C., Pinho S. T. (2009). Periodic forcing in a three-level cellular automata model for a vector-transmitted disease. *Physical Review E: Statistical, Nonlinear, and Soft Matter Physics*.

[14] Enduri M. K., Jolad S. (2018). Dynamics of dengue disease with human and vector mobility. *Spatial and Spatio-Temporal Epidemiology*.

[15] Wolfram S. (1994). *Cellular Automata and Complexity: Collected Papers*.

[16] Monteiro L. H. A., Chimara H. D. B., Berlinck J. G. C. (2006). Big cities: Shelters for contagious diseases. *Ecological Modelling*.

[17] Schimit P. H. T., Monteiro L. H. A. (2009). On the basic reproduction number and the topological properties of the contact network: An epidemiological study in mainly locally connected cellular automata. *Ecological Modelling*.

[18] Dias J. C. A., Monteiro L. H. A. Investigando a influência da distribuição espacial de mosquitos na propagação de doenças via autômato celular.

[19] Phaijoo G. R., Gurung D. B. (2017). Modeling Impact of Temperature and Human Movement on the Persistence of Dengue Disease. *Computational and Mathematical Methods in Medicine*.

[20] Cheng Y., Wang X., Pan Q., He M. (2017). Modeling the parasitic filariasis spread by mosquito in periodic environment. *Computational and Mathematical Methods in Medicine*.

[21] Parham P. E., Waldock J., Christophides G. K. (2015). Climate, environmental and socio-economic change: weighing up the balance in vector-borne disease transmission. *Philosophical Transactions of the Royal Society B: Biological Sciences*.

[22] Trpis M., Häusermann W., Craig G. B. (1995). Estimates of population size, dispersal, and longevity of domestic Aedes aegypti aegypti (Diptera: Culicidae) by mark-release-recapture in the village of Shauri Moyo in eastern Kenya.. *Journal of Medical Entomology*.

[23] Service M. W. (1997). Mosquito (Diptera: Culicidae) Dispersal - The Long and Short of It. *Journal of Medical Entomology*.

[24] Regis L., Monteiro A. M., De Melo-Santos M. A. V. (2008). Developing new approaches for detecting and preventing Aedes aegypti population outbreaks: Basis for surveillance, alert and control system. *Memórias do Instituto Oswaldo Cruz*.

[25] Wanji S., Mafo F. F., Tendongfor N. (2009). Spatial distribution, environmental and physicochemical characterization of Anopheles breeding sites in the Mount Cameroon region. *Journal of Vector Borne Diseases*.

[26] Lutambi A. M., Penny M. A., Smith T., Chitnis N. (2013). Mathematical modelling of mosquito dispersal in a heterogeneous environment. *Mathematical Biosciences*.

[27] Bartlett M. S. (1957). Measles Periodicity and Community Size. *Journal of the Royal Statistical Society. Series A (General)*.

